# LGR5 regulates osteogenic differentiation of human thoracic ligamentum flavum cells by Wnt signalling pathway

**DOI:** 10.1111/jcmm.17420

**Published:** 2022-06-06

**Authors:** Xiaoxi Yang, Chuiguo Sun, Xiangyu Meng, Guanghui Chen, Tianqi Fan, Chi Zhang, Zhongqiang Chen

**Affiliations:** ^1^ Department of Orthopedics Peking University Third Hospital Beijing China; ^2^ Central Laboratory Peking University International Hospital Beijing China

**Keywords:** endochondral ossification, LGR5, osteogenesis, thoracic ossification of the ligamentum flavum, Wnt signalling

## Abstract

Thoracic ossification of the ligamentum flavum (TOLF) is ectopic ossification of the spinal ligaments. Histologically, the development of TOLF can be described as the process of endochondral ossification. However, the underlying aetiology has not been completely clarified. In this investigation, the gene expression profile associated with leucine‐rich repeat‐containing G‐protein‐coupled receptors (LGR) and Wnt signalling pathway in the thoracic ligamentum flavum cells (TLFCs) of different ossification stages was analysed via RNA sequencing. We further confirmed the significant differences in the related gene expression profile by Gene Ontology (GO) enrichment analysis. LGR5 was first identified in primary human TLFCs during osteogenic differentiation. To evaluate the effect of LGR5 on osteogenic differentiation, LGR5 has been knocked down and overexpressed in human TLFCs. We observed that the knockdown of LGR5 inhibited the activity of Wnt signalling and attenuated the potential osteogenic differentiation of TLFCs, while overexpression of LGR5 activated the Wnt signalling pathway and increased osteogenic differentiation. Our results provide important evidence for the potent positive mediatory effects of LGR5 on osteogenesis by enhancing the Wnt signalling pathway in TOLF.

## 1 INTRODUCTION

Thoracic ossification of the ligamentum flavum (TOLF) is characterized by pathological ectopic ossification of the spinal ligaments, resulting in serious neurological dysfunction and server thoracic myelopathy due to stenosis of the spinal canal. The clinical features of TOLF are generally progressive and usually rarely responsive to conservative treatments except for surgical interventions.^1^, ^2^, ^3^ Recent studies have suggested the possible effects of mechanical stress,^4^, ^5^ inflammatory factors,^6^, ^7^ and genetic factors^8^, ^9^ that were contributed to the aetiology and pathogenesis of TOLF. However, the cellular and molecular mechanisms underlying are still not fully elucidated.

The development and progression of TOLF can be histologically described based on endochondral ossification.^10^ The ossified lesion in the spinal ligament contains both chondrocytes and osteoblasts, regulated by various transcriptional factors. Among those, leucine‐rich repeat‐containing G protein‐coupled receptors (LGRs), which belong to the leucine‐rich repeat‐containing G protein‐coupled 7‐transmembrane protein superfamily, are involved in the transmission of signals during homeostasis, bone remodelling and regenerative processes.^11^, ^12^ It has been noted that the expressions of various LGRs were involved in multiple bone‐specific cell types, including osteoblasts, osteoclasts and their progenitor populations. Recent studies have recognized LGR4, LGR5 and LGR6 as receptors of the R‐spondin proteins (RSPOs) family, which is comprised of four highly related secreted glycoproteins, RSPO1 to 4, respectively.^13^, ^14^, ^15^ By binding to RSPOs, LGR5 homologues were shown to modulate the activation of Wnt signalling pathway.^12^ The canonical Wnt signalling pathway is widely recognized as essential during the processes of bone formation and homeostasis. Furthermore, results from previous studies have also suggested Wnt signalling pathway might regulate the chondrogenesis and osteogenesis during the development of ossification of the spinal ligament.^16^, ^17^ However, the functional involvement of LGRs in the process of TOLF through Wnt signalling pathway is still needed to be addressed.

In the present study, we identified the LGRs that were specifically expressed in the thoracic ligamentum flavum cells (TLFCs) at different ossification stages by RNA sequencing based on micro‐computed tomography (micro‐CT) analysis, and further concluded that LGR5 promoted the osteogenesis in human TLFCs by activating Wnt signalling pathway.

## 2 MATERIALS AND METHODS

### 2.1 Patient specimens

The present study was approved by the Ethics Committee for Human Subjects of Peking University Third Hospital and performed in compliance with the Declaration of Helsinki (PUTH‐REC‐SOP‐06‐3.0‐A27, #2014003). Written Informed consent for the study was obtained from all patients. We retrospectively investigated the data of patients who have been diagnosed with TOLF from August 2015 to December 2018. The patients with cervical myelopathy, ossification of the posterior longitudinal ligament (OPLL), thoracic lumbar disc herniation, trauma, infection, deformity and other spinal disorders were excluded. A total of 28 patients diagnosed with TOLF who underwent decompressive laminectomy through posterior midline approach were enrolled in the study.

### 2.2 Micro‐CT evaluation and measurements

**FIGURE 1:**
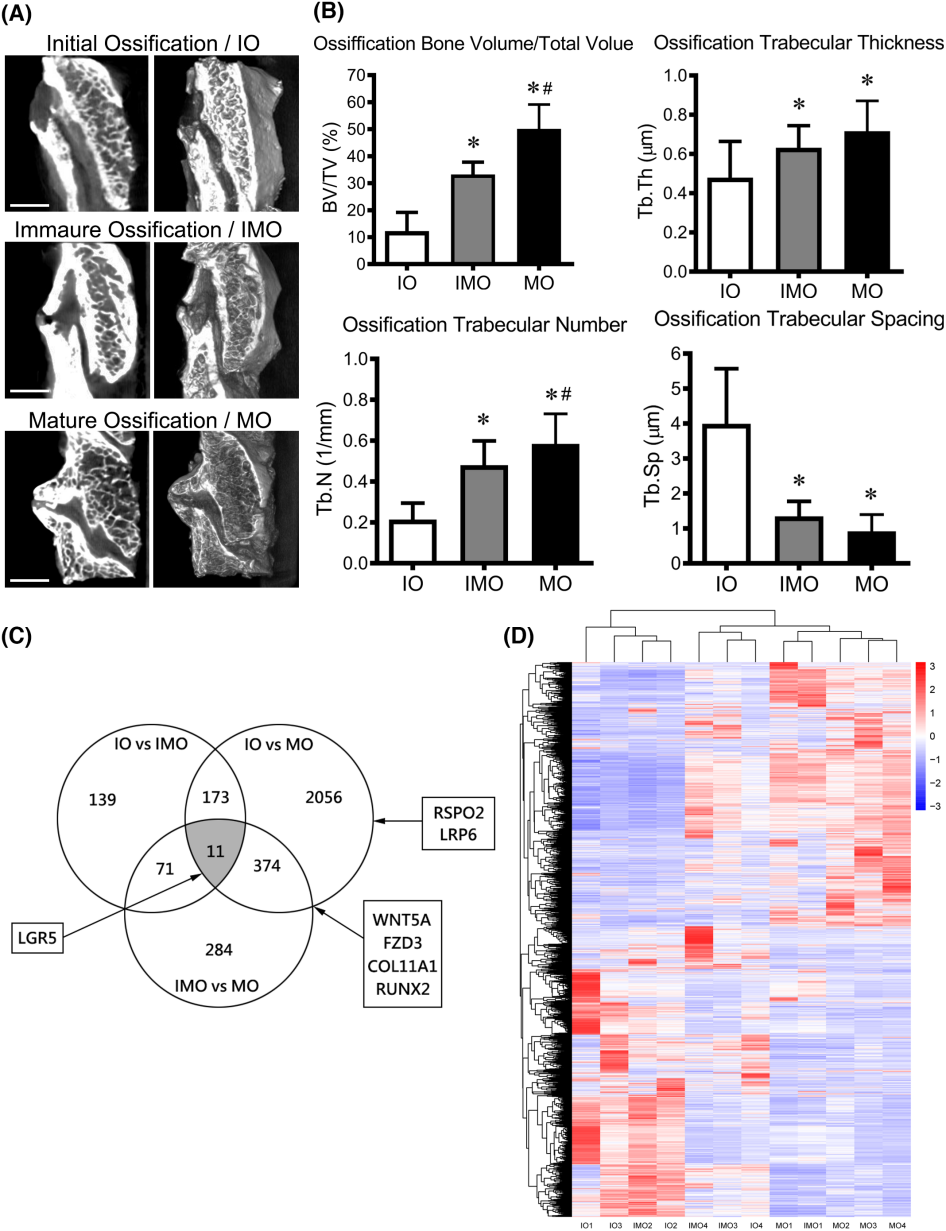
Morphological features and differentially expressed genes (DEGs) of thoracic ossification of the ligamentum flavum (TOLF) in different ossification stages. (A) micro‐CT images of different ossification stages of TOLF. Initial ossification (IO) of TOLF; immature ossification (IMO) of TOLF; mature ossification (MO) of TOLF. The scale bar represents 5 mm; (B) quantitative results of TOLF morphology; * *p* < 0.05 compared with IO group, # *p* < 0.05 compared with IMO group; (C) Strategy for the identification of DEGs; (D) Heat map of the hierarchical clustering of the different ossification stages of the DEGs

The lamina was completely resected by an ultrasonic bone curette, preserving the whole ossified mass intact to keep the integrity of the ligamentum flavum tissue. All lamina specimens were evaluated by micro‐CT using Inveon Acquisition Workplace (InveonTM, Siemens Medical Solutions) in alignment of the protocol we described previously.^18^ Three‐dimensional region of interest (ROI) was generated by manually depicted around the sites of ossified mass for subsequent analysis. We manually classified the ligamentum flavum to 3 different ossification stages of initial ossification (IO), immature ossification (IMO) and mature ossification (MO), based on the imaging features of micro‐CT. The ossification initiated at the attachment site of flavum ligament on lamina, low‐density hypertrophic ligamentum flavum was usually observed in IO stage. A bony shell enveloping the ligamentum flavum was then gradually formed from the attachment site on cranial and caudal lamina in IMO stage, with the decrease of normal structure. The bony shell structure increasingly thickened and fused in MO stage to form an ossified mass, similar to those observed in bone tissues, characterized by ligamentum flavum tissue been replaced by high‐density ossified structures (Figure 1A). The ossification degree was then categorized by calculating morphological parameters.

### 2.3 Haematoxylin & eosin and immunohistochemical (IHC) staining analysis

The resected ligament samples of different ossification stages were fixed in 10% buffered formalin and 10% formic acid for 1–4 weeks. After embedding in paraffin wax, the samples were deparaffinized and rehydrated, and then sectioned to 4 μm thick. Serial sections from different ossification stages were stained with haematoxylin & eosin following standard procedures. Representative sections for IHC staining were performed according to the manufacturer's manual as we described previously.^19^ The sections were incubated with the following mouse monoclonal Anti‐LGR5 primary antibody (1:100, Abcam) at 4°C after antigen retrieval with horse serum (ZSGB‐BIO), washed with PBS three times later and then treated with goat anti‐mouse IgG (ZSGB‐BIO) at room temperature for 30 min. To visualize the antibody binding reaction, sections were incubated with a diaminobenzidine (DAB) solution (ZSGB‐BIO), and nuclear counterstaining was performed with haematoxylin.

### 2.4 Cell culture and osteogenic differentiation

Cell culture was conducted in accordance with the protocol as described in our previous study.^9^ The ligament samples were collected during the patients' surgery procedures, washed with phosphate‐buffered saline (PBS) immediately. Based on the imaging features of micro‐CT analysis, the ligament samples were removed carefully from the ossified tissue under sterilized conditions. For IO stage, the samples away from the initiated ossified lesion at the attachment site of the ligament were harvested; for IMO stage, the samples adjacent to the ossified bony shell were used for the cell culture; for MO stage, the samples around the fusion site of the ossified mass were resected (Figure S1). The collected samples were minced into approximately 0.5 mm^3^ pieces, and then digested with 250 U/ml type I collagenase (Sigma‐Aldrich) at 37°C for 4 h and 0.25% trypsin (Gibco) at 37°C for 1 h. Next, the specimen was cultured in Dulbecco's Modified Eagle's medium (DMEM, Gibco) added 10% foetal bovine serum (Gibco), 100 U/ml penicillin and 100 μg/ml streptomycin at 37°C with 5% CO_2_. The osteogenic medium was supplemented with 50 μM ascorbic acid (Sigma‐Aldrich), 10 mM β‐glycerophosphate (Sigma‐Aldrich) and 10 nM dexamethasone (Sigma‐Aldrich) to induce the osteogenic differentiation. Cells from the third passage were used for the subsequent experiments.

### 2.5 RNA sequencing

Total RNA was extracted with TRIzol reagent (Invitrogen). RNA sequencing was performed by Novogene Bioinformatics Technology. Samples from four patients who had multiple‐level ossification were examined by RNA sequencing; the primary TLFCs of different ossification stages isolated from the same patient was served as a self‐control group. GO (http://www.geneontology.org/) enrichment analysis of differentially expressed genes (DEGs) was performed by the clusterProfiler R package (Version 6.8). GO terms with corrected *p* < 0.05 were considered significantly enriched by DEGs.

### 2.6 Quantitative real‐time polymerase chain reaction analysis

Quantitative real‐time polymerase chain reaction (qRT‐PCR) was performed using amiDETECTA Track miRNA qRT‐PCR Starter kit (RiboBio) and SYBR Green I (TaKaRa) according to the manufacturer's manual as we described previously.^7^ The relative gene expression levels were calculated using the 2^−ΔΔCt^ method. The primer sequences were listed below: LGR5: (F) 5'‐TGTTTCAGTGGCCTGCATTC‐3′ and (R) 5'‐AAGGTCATGGCTTGCAATGC‐3′; Wnt3a: (F) 5'‐TCCACGCCATTGCCTCAG 3′ and (R) 5'‐CACCATCCCACCAAACTCG‐3′; Wnt5a: (F) 5'‐GCGAGACGGCCTTCACAT‐3′ and (R) 5'‐TCCTTGGCAAAGCGGTAG‐3′; LRP6: (F) 5'‐GCCATTGCCATAGATTAC‐3′ and (R) 5′‐ TTGAGCCTTGTCACTTCT‐3′; FZD3: (F) 5'‐CACAAGATTCCGTTATCC‐3′ and (R) 5′‐ GGTACAGGCTTTATTATGAG‐3′; β‐catenin: (F) 5'‐ACGCTGCTCATCCCACTAAT‐3′ and (R) 5'‐AGTTCCGCGTCATCCTGATA‐3′; Runx2: (F) 5'‐GCACCGACAGCCCCAACTT‐3′ and (R) 5'‐CCACGGGCAGGGTCTTGTT‐3′; Osterix: (F) 5'‐GATGGCGTCCTCTCTGCTT‐3′ and (R) 5'‐TATGGCTTCTTTGTGCCTCC‐3′; ALP: (F) 5'‐AAGGACGCTGGGAAATCTGT‐3′ and (R) 5′‐ GGGCATCTCGTTGTCTGAGT‐3′; OCN: (F) 5′‐ CTCACACTCCTCGCCCTATT‐3′ and (R) 5'–GCCTGGGTCTCTTCACTAC‐3′; GAPDH: (F) 5′‐ CAGGAGGCATTGCTGATGAT‐3′ and (R) 5′‐ GAAGGCTGGGGCTCATTT‐3′.

### 2.7 Western blot analysis

A total of 50 μg protein was separated by SDS‐PAGE gel and transferred to PVDF membranes (Millipore) that were blocked with 5% bovine serum albumin (BSA), and then incubated with primary antibodies. Next, the samples were probed with appropriate HRP‐conjugated anti‐IgG and followed by detection with ChemiDoc XRS+ chemiluminescence system (Bio‐Rad Laboratories, Inc.). The following rabbit‐anti‐human monoclonal antibodies obtained from Abcam were used: anti‐LGR5 (1:1000); anti‐Runx2 (1:1000); anti‐Osterix (1:2000); anti‐ALP (1:2000); anti‐OCN (1:500); anti‐Wnt3a (1:1000); anti‐Wnt5a (1:1000); anti‐LRP6 (1:1000); anti‐FZD3 (1:1000); anti‐β‐cantenin (1:1000) and anti‐GAPDH (1:2500).

### 2.8 Alkaline phosphatase activity assay

After 7 days of osteogenic induction, TLFCs (1 × 10^5^ /well) were seeded in 6‐well plates and cultured in osteogenic medium for 7 days. The alkaline phosphatase (ALP) activity was assessed by an ALP activity staining kit (GENMED Scientifics), in accordance with the manufacturer's protocol recommendations.

### 2.9 Lentiviral transfection of thoracic ligamentum flavum cells

The lentivirus‐based vectors for knockdown (short hairpin RNA, shRNA) and overexpression of LGR5 were ordered from Hanbio. The sequences of the shRNA‐LGR5 (Lv‐shLGR5) were listed as follows: top strand: 5'‐CACCGCTCAGAATAATCAGCTAAGCTCGAGCTTAGCTGATTATTCTGCAGC‐3′, bottom strand: 5'‐AAAAGCTGCAGAATAATCAGCTAAGCTCGAGCTTAGCTGATTATTCTGCAGC‐3′. A negative control shRNA (NC) with non‐complementary sequences was used as control. Lentivirus vectors encoding the whole length of human LGR5 (Lv‐LGR5) was transfected in TLFCs for LGR5 overexpression. Empty lentivirus vectors were used as negative control (Lv‐NC). The primary TLFCs of initial ossification stage were seeded in a 100 mm dish with a density of 5 x 10^5^ /well. The transfection mix with a solution of 100 μl consisting of 5 x 10^5^ PFU lentivirus was added to the dish on day 2, and then, the cells were incubated in 5% CO_2_ at 37°C for 7 days. Stable knockdown and overexpression of LGR5 in the TLFCs were verified by both qRT‐PCR and Western blot analysis.

### 2.10 Inhibitor of Wnt response 1 (IWR‐1) inhibitory assay

Thoracic ligamentum flavum cells at the initial ossification stage with or without stable LGR5 overexpression were treated with IWR‐1 (10μM, Sigma‐Aldrich) for 48 h during culture in osteogenic differentiation medium. TLFCs without the transfection of lentivirus were served as blank group (Blank).

### 2.11 Statistical analysis

All values were presented as mean ± SD. Each experiment was performed in triplicate. Comparisons between two groups were assessed by two‐tailed *t*‐test, and comparisons among three or more groups were analysed using one‐way analysis of variance (anova). Data were evaluated by SPSS 24.0 and statistical significance was defined as *p* < 0.05.

## 3 RESULTS

### 3.1 Morphological characteristics of ossified ligamentum flavum in thoracic ligamentum flavum cells

A total of 56 samples were collected from 28 TOLF patients (14 male and 14 female) with a mean age of 54.74 ± 9.25 years. Three different ossification stages of TOLF including initial ossification (IO), immature ossification (IMO) and mature ossification (MO) were observed on micro‐CT images (Figure 1A). IO of the ligamentum flavum was detected only at the attachment point, without bony shell, whereas IMO and MO were observed either on the bony shell or the ossified mass along the lamina. Of all samples, 11 were at the IO stage, 21 at the IMO stage and 24 at the MO stage. The ossification stages were identified by morphological measurements in ROI (Figure 1B).

### 3.2 DEGs of TLFCs in different ossification stages established via RNA‐sequencing

Totally, a number of 3108 DEGs were identified in the IO‐IMO, IMO‐MO and IO‐MO groups of TOLF (Figure 1C). To identify the DEGs that contributed to the osteogenic feature of the TLFCs of different ossification stage, we examined the overlaps of the DEGs expression in three groups, where 11 overlapping DEGs were identified, including LGR5, secreted phosphoprotein 1 (SPP1) and fibulin‐2 (FBLN2). Higher expression of LGR5 was observed in IO group compared with IMO (>1.96 fold) and MO (>17.19 fold) groups. A total of 374 DEGs were shared exclusively between the IO‐MO and IMO‐MO groups, where Wnt5a (<0.36 fold, IO vs. MO), FZD3 (<0.32 fold, IO vs. MO), COL11A1 (<0.20 fold, IO vs. MO) and Runx2 (<0.27 fold, IO vs. MO) were detected. Meanwhile, RSPO2 (>7.37 fold) and LRP6 (<0.58 fold) were also identified as the DEGs in the IO‐MO group together with other osteogenesis‐related factors, including Osterix, Runx2 and ALP. The distinctive DEGs expressed in the TLFCs of different ossification stages are presented in the heat map (Figure 1D). The heat map with showname of DEGs was submitted as Supporting Information (Data S1). The full lists of DEGs were shown in Table S1‐3.

Gene Ontology analysis was performed to further investigate the biological functions of the identified DEGs in the TLFCs. The results indicated that most of the DEGs were enriched in Wnt signalling pathway and ossification (Table 1).

**TABLE 1:** GO enrichment analysis results of the DEGs

Category	ID	Description	*p* value
IO vs. MO	GO:0060070	canonical Wnt signalling pathway	0.004
	GO:0030278	regulation of ossification	0.007
	GO:0016055	Wnt signalling pathway	0.015
	GO:0035988	chondrocyte proliferation	0.036
IMO vs. MO	GO:0001503	Ossification	0.000
	GO:0030282	Bone mineralization	0.007
	GO:0016055	Wnt signalling pathway	0.024
IO vs. IMO	GO:0001503	Ossification	0.375
	GO:0090263	Regulation of canonical Wnt signalling pathway	0.387
	GO:0001958	Endochondral ossification	0.550

Abbreviations: DEGs, differentially expressed genes; GO, Gene Ontology; IO, initial ossification; IMO, immature ossification; MO, mature ossification.

### 3.3 Expression of LGR5 at different ossification stages in TOLF Sections

**FIGURE 2:**
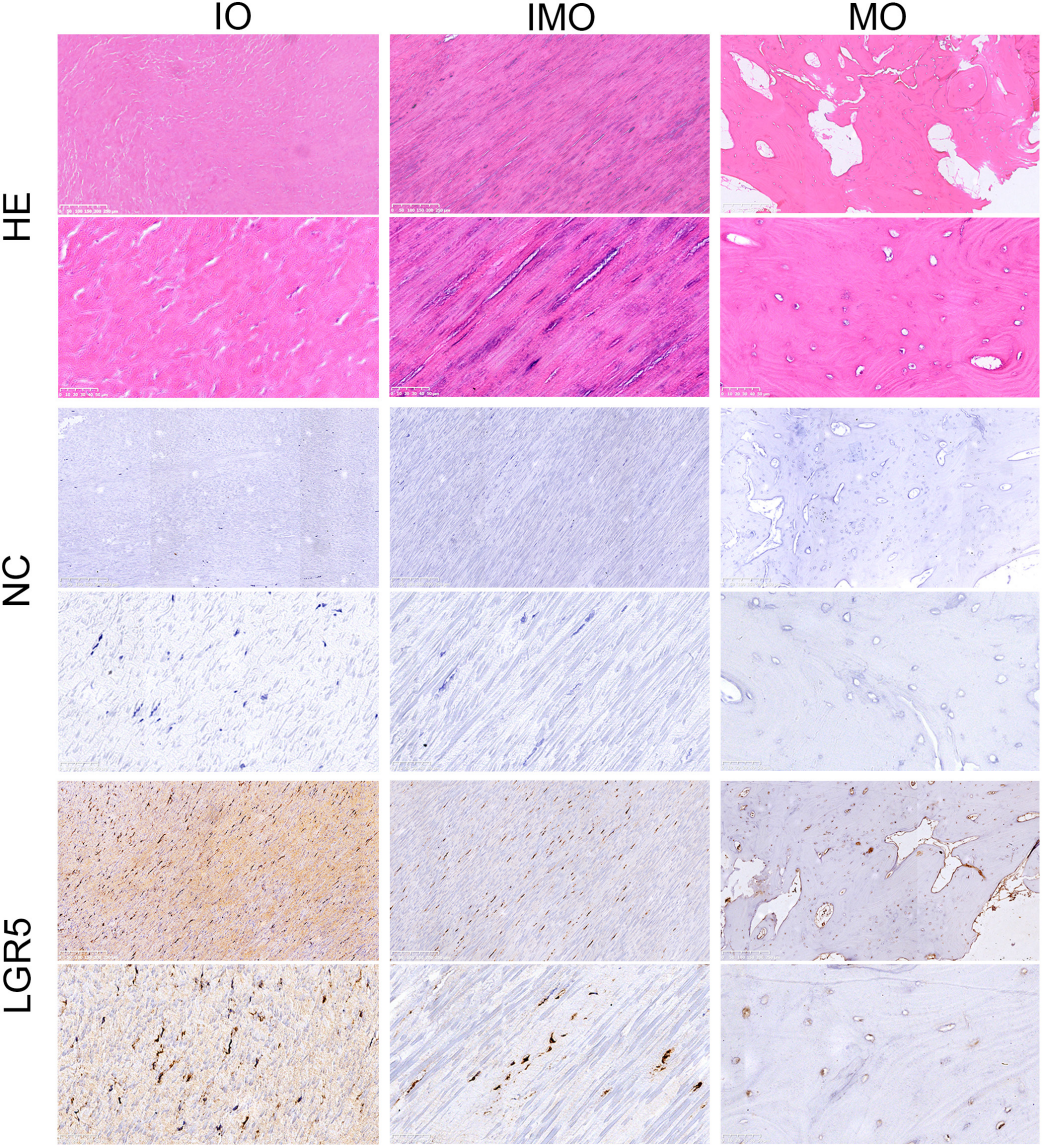
Differentially expressed genes (DEGs) of thoracic ligamentum flavum cells (TLFCs) in different ossification stages via RNA‐sequencing. Representative images of haematoxylin and eosin staining and immunohistochemical (IHC) staining for LGR5 in ligament flavum samples of different ossification stages. Scale bar represents 250 μm in low‐power field and 50 μm in high‐power field

The expression of LGR5 in the fibroblast‐like cells was further confirmed by IHC analysis. The strongly expression of LGR5 was observed in the regularly arranged elastic fibres with hyperplastic collagen fibres away from the ossified lesion in IO stage, then decreased in the compacted collagen fibres with calcification adjacent to the ossified lesion in IMO stage. In MO stage, the expression of LGR5 was further lowered in the calcified area with disappearance of fibre bundles at the fusion site of ossified mass (Figure 2).

### 3.4 Expression level of LGR5 and Wnt signalling in primary TLFCs

**FIGURE 3:**
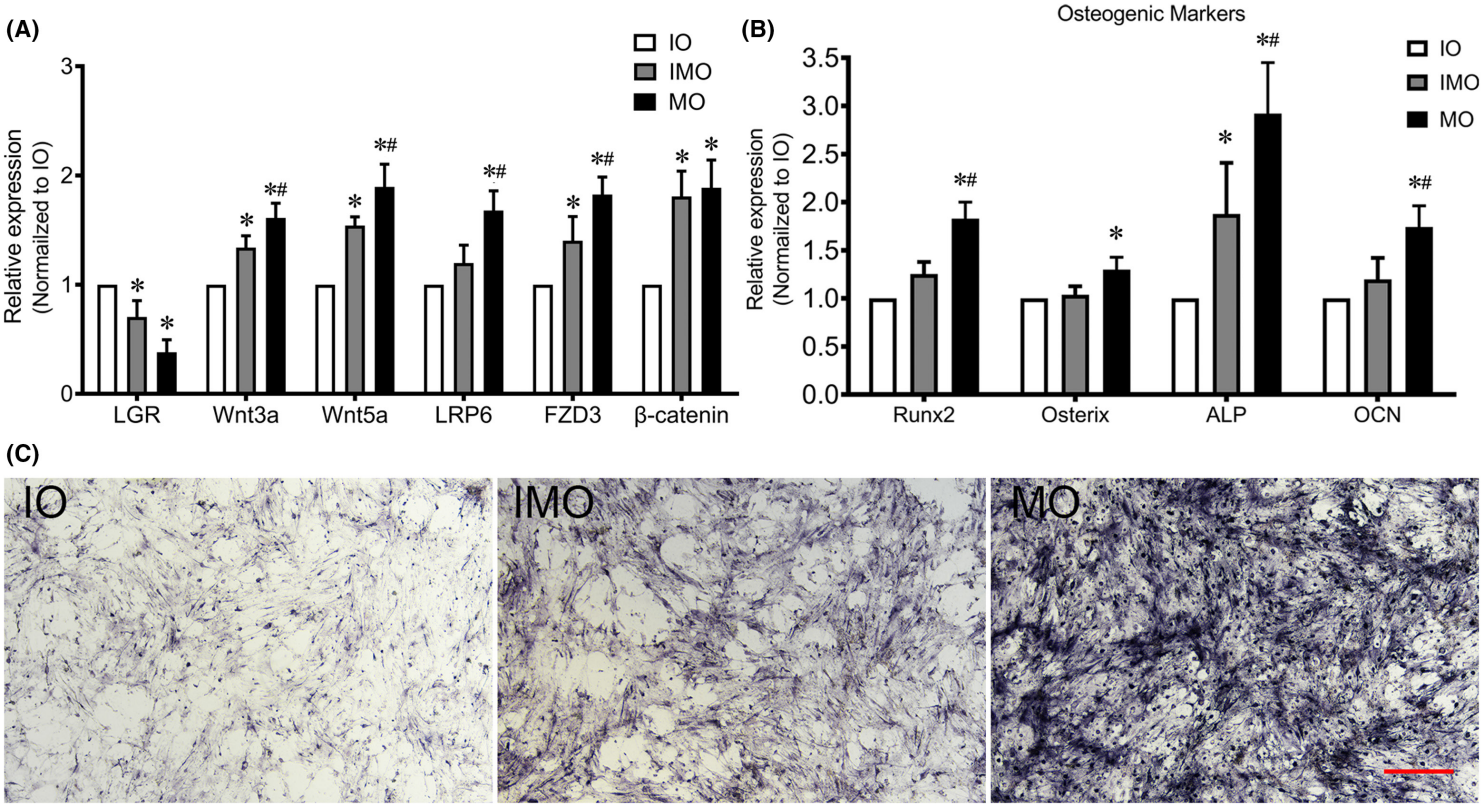
Expression level of the genes in the primary thoracic ligamentum flavum cells (TLFCs) at different ossification stages. (A and B) the expression of LGR5, Wnt related genes and osteogenic markers was assessed by qRT‐PCR; *p* < 0.05 vs. IO group, #*p* < 0.05 vs. IMO group; (C) ALP staining of the TLFCs at different ossification stages; the scale bar represents 500 μm

To confirm the functional relevance of the DEGs, we compared the mRNA expression level of LGR5, Wnt signalling activity and osteogenic capacities of the primary TLFCs of different ossification stages. The cells at the IO stage had a higher expression level of LGR5 than those at the IMO and MO stages. The expression of LGR5 decreased during ossification, on the contrary, Wnt3a, Wnt5a, LRP6, FZD3 and β‐catenin were significantly increased (Figure 3A). The elevated expression of the osteogenic markers was observed in the TLFCs of different ossification stages as well (Figure 3B). ALP staining was performed on Day 7 (Figure 3C). Our findings were consistent with the RNA‐sequencing data.

### 3.5 LGR5 knockdown inhibits the Wnt signalling and osteogenic differentiation in primary TLFCs

**FIGURE 4:**
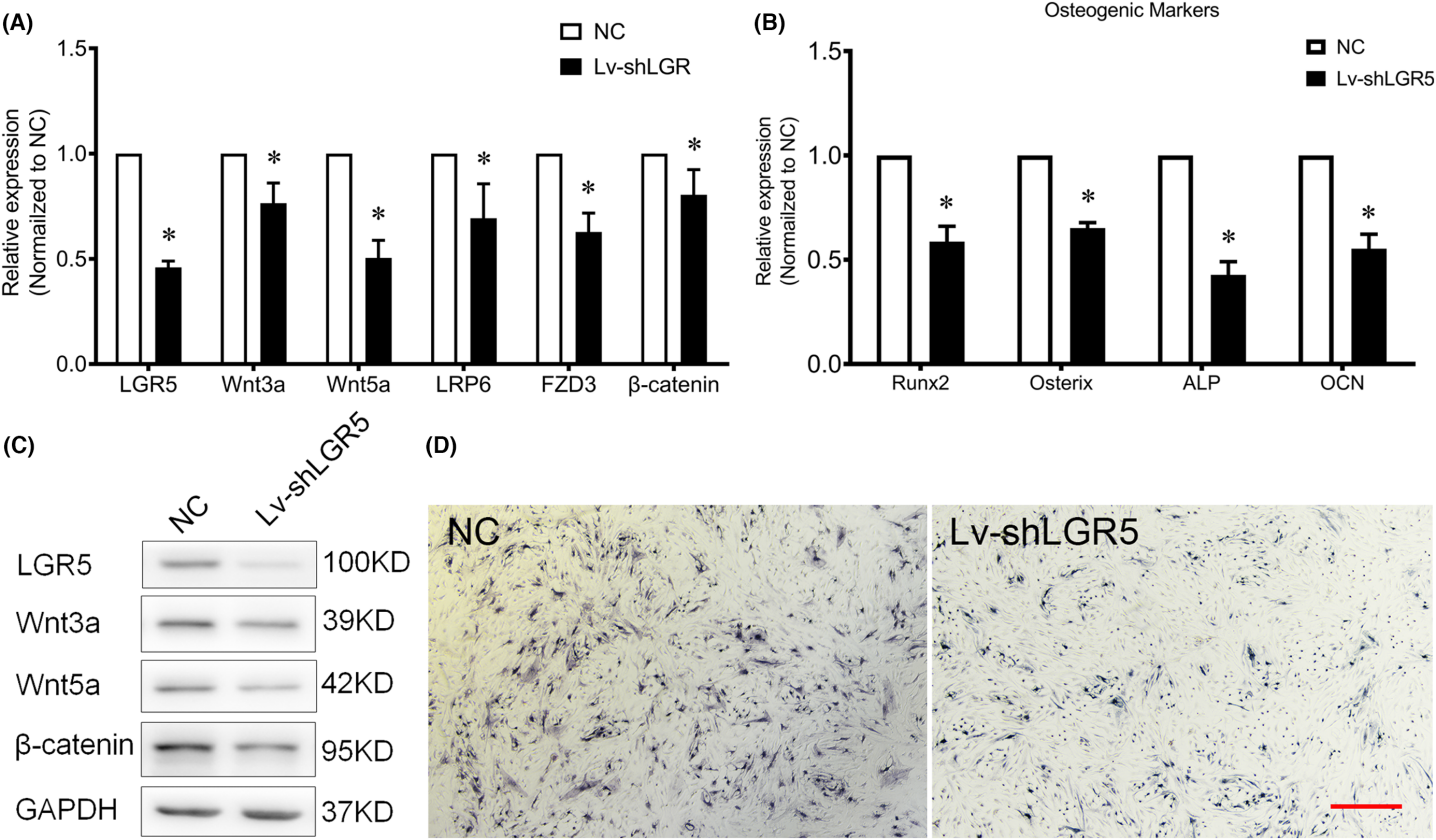
LGR5 knockdown inhibits the Wnt signalling and osteogenic differentiation of the thoracic ligamentum flavum cells (TLFCs). (A and B) the effects of the LGR5 knockdown on mRNA levels of Wnt related genes and osteogenic markers were evaluated by qRT‐PCR after Lv‐shLGR5 transfection, *p < 0.05 vs. NC group; (C) the effects of the LGR5 knockdown on the protein levels of Wnt related genes determined by Western blot analysis; (D) ALP staining following the LGR5 knockdown; the scale bar represents 500 μm

To validate whether LGR5 is involved in osteogenic differentiation, we knocked down LGR5 by delivering specific Lv‐shRNA. The significant reduced expression of LGR5 in the TLFCs was then confirmed. Similarly, the mRNA and protein expression levels Wnt signalling were also found to be decreased (Figure 4A,C), as well as the osteogenic markers (Figure 4B). Attenuated ALP activity was detected in the LGR5 knockdown cells as well (Figure 4D). These results suggested that the LGR5 knockdown inhibited the osteogenic differentiation of the TLFCs.

### 3.6 LGR5 overexpression enhances the Wnt signalling and osteogenic differentiation in primary TLFCs

**FIGURE 5:**
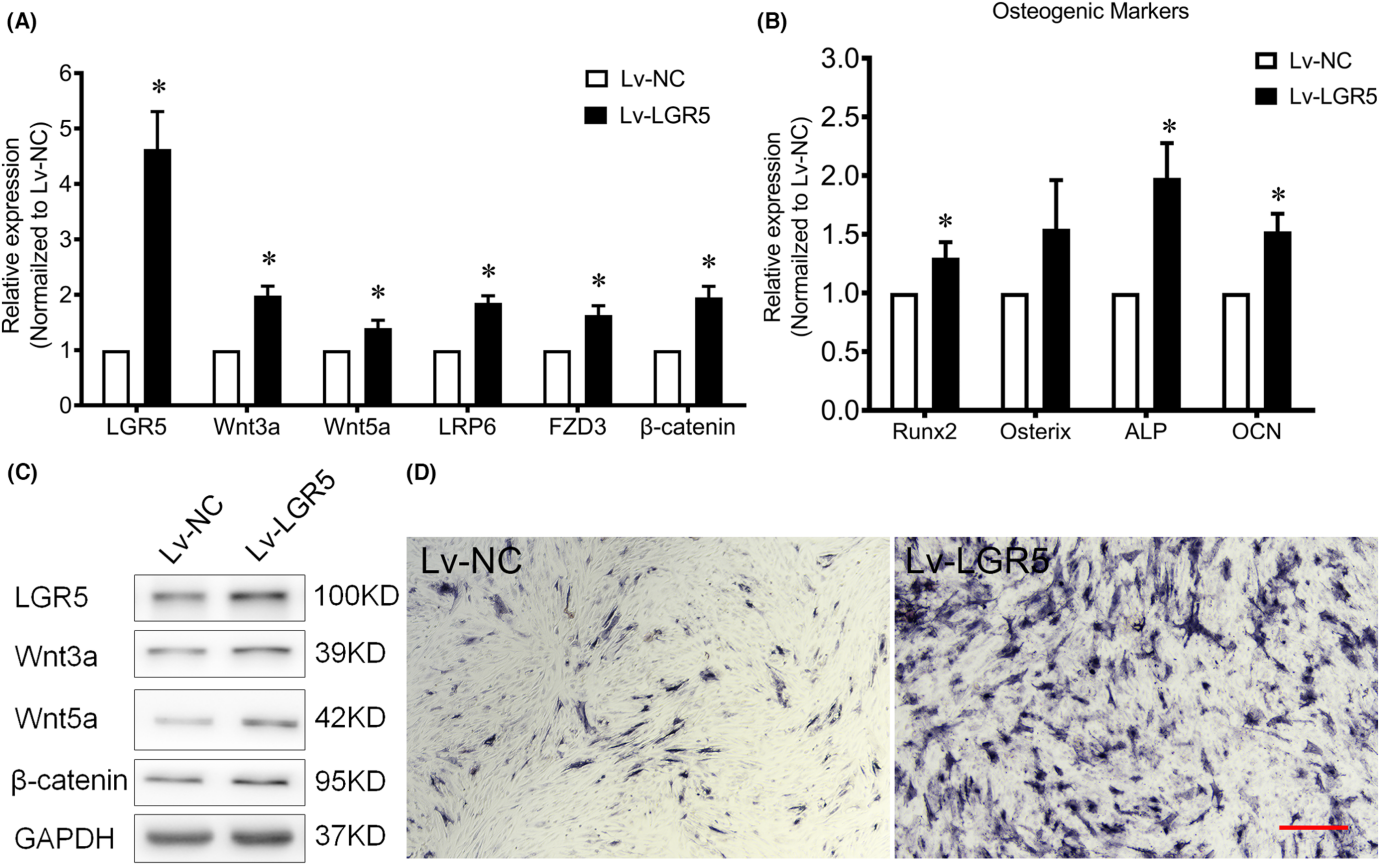
LGR5 overexpression enhances the Wnt signalling and osteogenic differentiation of the thoracic ligamentum flavum cells (TLFCs). A and B, the effects of the LGR5 overexpression on mRNA levels of Wnt related genes and osteogenic markers were evaluated by qRT‐PCR after Lv‐LGR5 transfection, *p < 0.05 vs. Lv‐NC group; (C) the effects of the LGR5 overexpression on the protein levels of Wnt related genes measured by Western blot analysis; (D) ALP staining following the LGR5 overexpression; the scale bar represents 500 μm

To evaluate the effects of the overexpression of LGR5 on the osteogenic differentiation and Wnt signalling pathway, Lv‐LGR5 was utilized to transfect the primary TLFCs at the initial ossification stage for 7 days. As visible in Figure 5A–D, the overexpression of LGR5 strengthened the expression of Wnt related gene and the osteogenic markers. These results indicated that the overexpression of LGR5 would activate the Wnt signalling pathway and promote of the osteogenic differentiation potential.

### 3.7 Inhibition of Wnt signalling pathway attenuates the osteogenic differentiation induced by LGR5 overexpression in primary TLFCs

**FIGURE 6:**
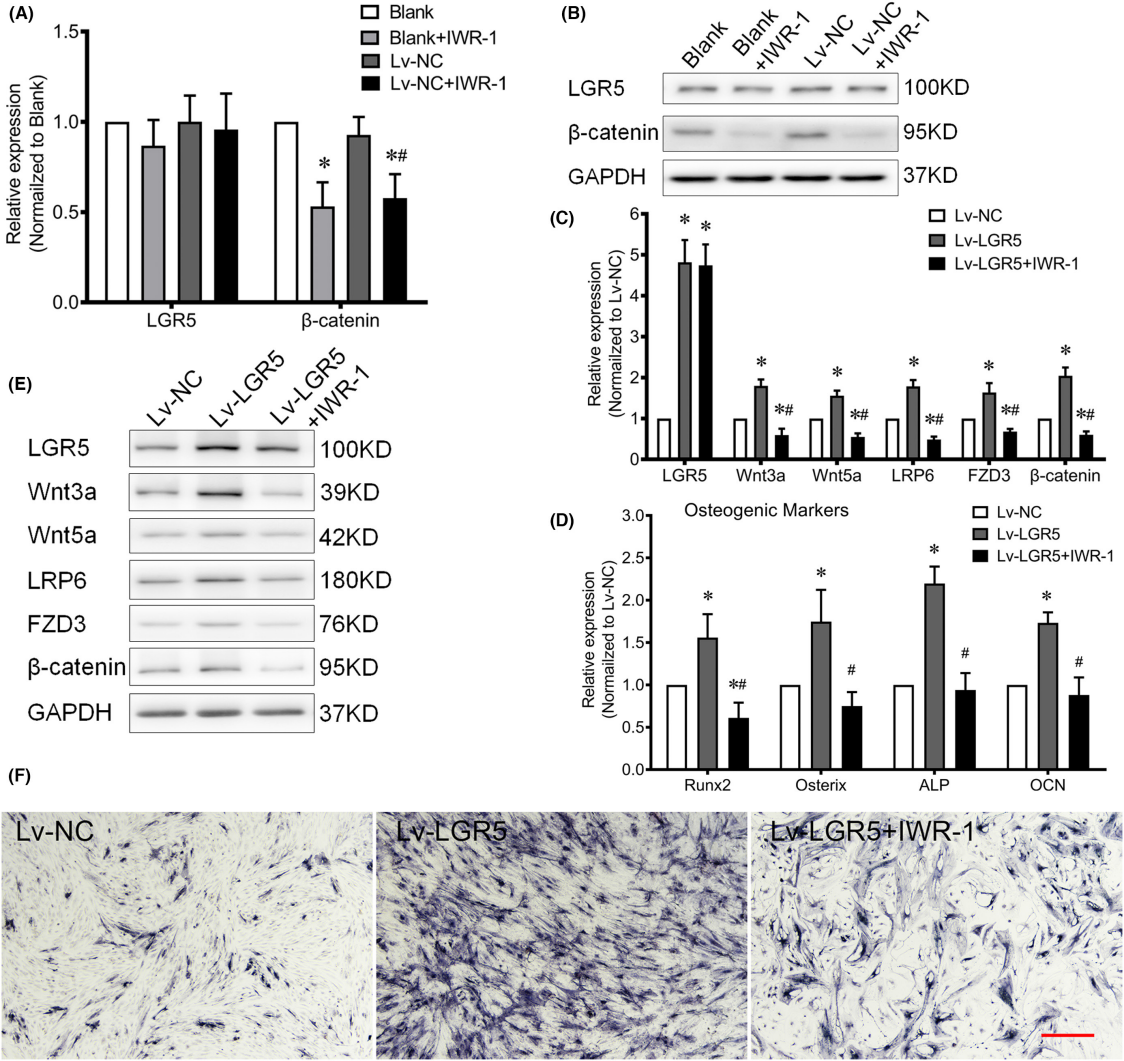
IWR‐1 inhibits the osteogenic differentiation of the TLFCs with stable expression of LGR5. (A) the effects of IWR‐1 on LGR5 and β‐catenin expression levels in the primary thoracic ligamentum flavum cells (TLFCs) without LGR5 overexpression were measured via qRT‐PCR; *p < 0.05 vs. blank group, # *p* < 0.05 vs. Lv‐NC group; (B) the effect of IWR‐1 on the LGR5 and β‐catenin protein levels; (C and D) the effects of IWR‐1 on LGR5, Wnt related genes and osteogenic markers expression levels were analysed after Lv‐LGR5 transfection via qRT‐PCR; *p < 0.05 vs. Lv‐LGR5 group; (E) the effects of IWR‐1 on the protein levels of LGR5 and Wnt related genes determined via Western blot analysis; F, ALP staining following the IWR‐1 treatment; the scale bar represents 500 μm

The tankyrase inhibitor IWR‐1 was used to further determine whether Wnt signalling pathway mediates the enhanced osteogenic differentiation of LGR5. The expression level of β‐catenin in blank group and Lv‐NC group was significantly attenuated with IWR‐1. But the IWR‐1‐treated TLFCs without stable LGR5 overexpression had insignificant changes in their mRNA and protein levels of LGR5 compared with blank group and Lv‐NC group (Figure 6A,B). No significant different expression was observed between blank group and Lv‐NC group. LGR5‐overexpressing TLFCs in IO stage were then treated with IWR‐1, and found that the enhanced expression of Wnt related genes and osteogenic markers induced by LGR5 overexpression was mitigated by introducing of IWR‐1 (Figure 6C–F). Taken together, LGR5 promotes the osteogenesis of TLFCs in a Wnt signalling‐dependent manner.

## 4 DISCUSSION

Thoracic ossification of the ligamentum flavum is a rare spinal ligament ectopic ossification that usually causes severe neurological dysfunction with poor prognosis, attributed to complex underlying pathology and associated factors. The progression of TOLF can be characterized as an endochondral ossification process. The histological process initiated with chondroid metaplasia and infiltration of fibroblast‐like mesenchymal cells into the ligamentum matrix, followed by gradual maturation and the synthesis of extracellular matrix under the mediation of various transcription factors.^10^ The crucial role of Wnt signalling pathway in the regulation of bone maintenance and homeostasis has been recognized.^16^ However, the relationship between upstream signal transduction and spinal ligament degeneration has not yet been clarified. In our present paper, we report for the first time that LGR5 is a potent positive mediator of osteogenesis in human TLFCs by activating the Wnt signalling pathway.

Based on the different morphological characteristics of TOLF analysed by micro‐CT, the DEGs in the primary TLFCs of different ossification stages were identified by RNA‐sequencing, including LGR5, RSPO2, Wnt5a, LRP6, FZD3, COL11A1 and Runx2. The emerging role of LGRs in Wnt signalling‐associated adult stem cell markers in osteogenic differentiation and skeletal regeneration has already been indicated in previous studies.^20^, ^21^ It has been proved that LGR5 was involved in the formation of articular cartilage, cruciate ligament and meniscus of embryonic mice during skeletal development.^20^ In addition, enhanced expression of LGR5 and RSPO2 was detected in chondrocytic differentiation of human chondrocytes in advanced stage of osteoarthritis, while elevated expression of LGR6 and RSPO1 was observed in osteogenic differentiation of SaOS‐2 cells.^21^ Using RNA‐sequencing analysis, we differentiated the expression of LGR5 and RSPO2 in the TLFCs of different stages and recognized the importance of LGR5 as a regulator facilitating the differentiation of proliferating chondrocytes in endochondral ossification. We found that LGR5 might initiate the phenotype transdifferentiation of TLFCs by binding RSPO2, marked the start of endochondral ossification. Going towards the advanced stage of ossification, the expression of LGR5 was then down regulated. Our IHC results further verified that the expression of LGR5 gradually decreased during the process from initial to mature ossification as the morphology and structure of the TLFCs transformed from fibroblast‐like cells to chondroid‐ or osteoblast‐like cells. The ossification process was henceforth promoted mainly by the early‐activated chondrogenic markers (e.g. COL11A1) and osteogenic markers (e.g. Runx2, ALP). Furthermore, the expression levels of Wnt5a, LRP6 and FZD3were promoted during the osteogenic maturation process, which was consistent with the results of previous studies exploring the mechanism of Wnt5a and LRP6 in osteoblastogenesis regulation and Wnt signalling pathway.^22^, ^23^, ^24^ Another investigation also reported that decreased DNA methylation of Wnt5a might promote the osteogenesis in ossified spinal ligaments.^25^ In according with prior reports, our findings recognized LGR5 as a potential regulator promoting the osteogenesis of TLFCs at the initial ossification stage by the Wnt signalling pathway. Subsequent experiments were therefore performed on TLFCs at IO stage to further explore the effect of LGR5. Meanwhile, our results suggested the potential process of transdifferentiation from chondrocytic proliferation to osteoblastic differentiation during the endochondral ossification of TOLF.

LGR5 is a G protein‐coupled 7‐transmembrane protein belonging to the rhodopsin family, and is a potent Wnt target complex served as a significant modulator of Wnt signalling pathway.^12^, ^26^, ^27^, ^28^ LGR5‐overexpression enhances the osteogenesis of mesenchymal stem cells by promoting the expression of pERK1/2 and β‐catenin in Wnt/ERK signalling pathways. Attenuated osteogenic capacity was induced by silencing the LGR5 expression, which possibly attributed to increased mitochondrial fragmentation and reduced mitochondrial biogenesis.^29^ In an LGR‐null model, LGR5 knock‐out mice were nonviable after birth due to cleft palate‐like phenotype, indicating that LGR5 was contributed to the craniofacial formation.^30^ LGR5 was also found to be expressed in the periodontal ligament and to be critically involved in the skeletal remodelling of the alveolar bone.^31^ Our findings confirmed that LGR5 knockdown inhibited the activity of the Wnt signalling and prevented the latent osteogenic differentiation of the TLFCs at the initial stage, whereas the opposite effects were demonstrated if LGR5 was overexpressed. It is worth noted the significance of Wnt signalling pathway in the regulation of bone homeostasis. To investigate whether the activation of Wnt signalling was required for enhanced osteogenic differentiation of LGR5 overexpressed TLFCs, we blocked the Wnt signalling pathway using the inhibitor IWR‐1. The results confirmed that the enhanced osteogenic differentiation induced by LGR5 overexpression was mitigated by IWR‐1 intervention. Furthermore, the downregulation of Wnt signalling via IWR‐1 had no effect on the expression of LGR5 in the TLFCs. Therefore, the activation of Wnt signalling triggered the osteogenic differentiation of TLFCs, which was mediated by LGR5.

RSPOs were first discovered as secreted Wnt signalling agonists and were identified as related ligands of LGRs. Previous studies showed that LGRs had a high affinity for binding to RSPOs, activating the Wnt/β‐catenin and Wnt/PCP signalling pathways.^12^, ^15^ Both RSPO1 and RSPO2 were found to promote the osteoblast differentiation in synergy by Wnt signalling pathway in vitro.^32^, ^33^, ^34^ The causal single nucleotide polymorphism (rs374810) located in RSPO2 was identified as a susceptibility gene to mediate chondrogenesis by activating Wnt signalling in OPLL.^17^, ^35^ Data from the MC3T3‐E1 cell line again showed that RSPO2 promoted osteoblast formation through activating the canonical Wnt/β‐catenin signalling, and LGR5 was involved in the RSPO2 mediated β‐catenin stabilization.^36^ In our study, the elevated expression of RSPO2 and LGR5 at the initial ossification stage of TLFCs was verified. It has been reported that LGRs could regulate both canonical and non‐canonical pathway by interacting with RSPO and ZNRF3/RNF43, preventing the degradation of FZDs, thus stimulated both canonical and non‐canonical Wnt ligands at the initiation stage.^37^ In RNA sequencing analysis of primary cells of different ossification stages, we found that the expression of Wnt3a and Wnt5a were consistent with Wnt pathway factors (e.g. LRP6 and FZD3) throughout the ossification process, suggesting a synergistic effect that both Wnt canonical and non‐canonical pathway were involved. Taken together, our findings indicated that RSPO2 had a critical role in LGR5‐mediated osteogenesis by inducing Wnt signalling activation. However, the underlying molecular mechanisms of these processes still need to be addressed. Further studies are recommended to shed light on whether LGR5 regulates the Wnt signalling pathway directly through RSPO2.

In conclusion, our present study demonstrated the importance of LGR5 in osteogenic differentiation of TLFCs, and provide the first evidence that LGR5 be a positive regulator of osteogenesis in TOLF by activating Wnt signalling pathway.

## AUTHOR CONTRIBUTIONS

**Xiaoxi Yang:** Conceptualization (equal); data curation (equal); investigation (equal); methodology (equal); software (equal); writing – original draft (lead); writing – review and editing (lead). **Chuiguo Sun:** Data curation (equal); methodology (equal); resources (lead); writing – review and editing (equal). **Xiangyu Meng:** Data curation (equal); funding acquisition (equal); investigation (equal); methodology (equal); software (equal). **Guanghui Chen:** Data curation (equal); investigation (equal); resources (equal); software (equal). **Tianqi Fan:** Data curation (equal); formal analysis (equal); software (equal). **Chi Zhang:** Data curation (equal); methodology (equal); resources (equal); supervision (equal). **Zhongqiang Chen:** Conceptualization (lead); funding acquisition (lead); project administration (equal); resources (equal); supervision (lead); writing – review and editing (lead).

## CONFLICT OF INTEREST

The authors declare no conflicts of interest.

## Supporting information


Data S1

Figure S1

Table S1

Table S2

Table S3

## Data Availability

Data are available from the corresponding author on request.
