# Supplementary figures and images for: LGR5 regulates osteogenic differentiation of human thoracic ligamentum flavum cells by Wnt signalling pathway
Source: J Cell Mol Med. 2022 Jun 6;26(14):3862–72. doi: 10.1111/jcmm.17420 (PMC9279595; doi:10.1111/jcmm.17420)

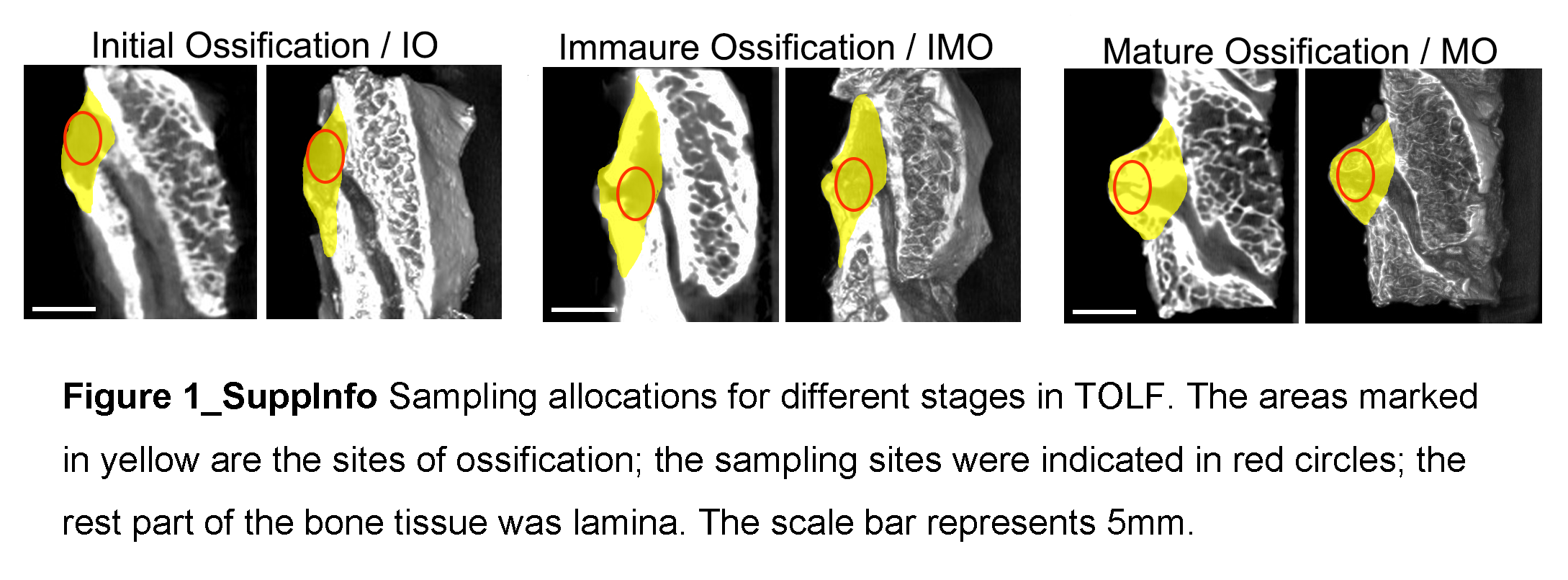

Supplement: Supplementary file 2 — Figure S1 [file JCMM-26-3862-s004.zip › JCMM_17420_Figure 1_SuppInfo.tif]
