# Supplementary material for: LGR5 regulates osteogenic differentiation of human thoracic ligamentum flavum cells by Wnt signalling pathway
Source: J Cell Mol Med. 2022 Jun 6;26(14):3862–72. doi: 10.1111/jcmm.17420 (PMC9279595; doi:10.1111/jcmm.17420)
Supplement: Supplementary file 1 — Data S1 [file JCMM-26-3862-s002.pdf]

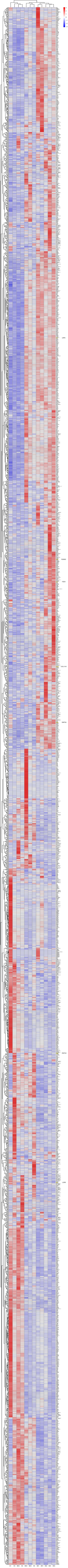

Document 1_SuppoInfo Heat map of DEGs with highlights of LGR5 and other related genes. LGR5, RSPO2, WNT5A, LRP6, FZD3, COL11A1, RUNX2 were shown in yellow highlights.
